# Probabilistic projections of El Niño Southern Oscillation properties accounting for model dependence and skill

**DOI:** 10.1038/s41598-022-26513-3

**Published:** 2022-12-22

**Authors:** Roman Olson, Soong-Ki Kim, Yanan Fan, Soon-Il An

**Affiliations:** 1 Irreversible Climate Change Research Center, Yonsei University, Seoul, South Korea; 2 Institute of Industrial Science, University of Tokyo, Kashiwa, Japan; 3 Department of Atmospheric Sciences, Yonsei University, Seoul, South Korea; 4 Data61, CSIRO, Sydney, Australia; 5 Division of Environmental Science and Engineering, Pohang University of Science and Technology (POSTECH), Pohang, South Korea

**Keywords:** Projection and prediction, Physical oceanography, Statistics

## Abstract

The El Niño – Southern Oscillation (ENSO) is a dominant mode of global climate variability. Nevertheless, future multi-model probabilistic projections of ENSO properties have not yet been made. Main roadblocks that have been hindering making these projections are climate model dependence and difficulty in quantifying historical model performance. Dependence is broadly defined as similarity between climate model output, assumptions, or physical parameterizations. Here, we propose a unifying metric of relative model performance, based on the probability density function (PDF) of ENSO paths. This metric is applied to assess the overall skill of Climate Model Intercomparison Project phase 6 (CMIP6) climate models at capturing ENSO. We then perform future multi-model probabilistic projections of changes in ENSO properties (from years 1850–1949 to 2040–2099) under the shared socioeconomic pathway scenario SSP585, accounting for model skill and dependence. We find that future ENSO will likely be more seasonally locked (89% chance), and have a longer period (67% chance). Yet, the jury is still out on future ENSO amplification. Our method reduces uncertainty by up to 37% compared to a simple approach ignoring model dependence and skill.

## Introduction

The El Niño – Southern Oscillation (ENSO) is a dominant pattern of global climate variability^1–7^. It is characterized by quasi-periodic warming (El Niño) and cooling (La Niña) of the eastern equatorial Pacific sea surface temperatures (SSTs), which lead to associated changes in the tropical climate. ENSO’s extended reach stems out of its ability to regulate climate across the globe through its teleconnections^8,9^.

Recent history is rife with examples of ENSO’s powerful impacts. The 1997–1998 El Niño is the strongest event on record, and it was associated with destructive floods, droughts, fires, extreme heat, damage to crops, as well as a hantavirus outbreak^10–12^. Other major El Niño and La Niña events were also associated with devastating damages. For example, the 1982–1983 El Niño caused 40 times more rain than usual in Peru and Ecuador, while Indonesia, Australia and Philippines suffered from drought^10,13,14^. Anomalous fatal fires were reported in Australia^10,13^. At the same time, strong waves off the shore of California have led to 14 casualties, urgent evacuation of 15,000 people, and resulted in $265 million in damages^10^. Moreover, Africa suffered from devastating droughts causing starvation to death^10,14^. At the same time a powerful malaria outbreak rampaged through Peru and South America^13^. The total damages from the 1982–1983 El Niño were estimated at $8.1 billion^10^.

Long-term ENSO projections are typically made using climate models – complex pieces of computer code which solve equations of atmospheric and oceanic dynamics and thermodynamics on a grid, while also typically including other relevant climate components such as vegetation, sea ice, etc.^15^. However, there is a lack of statistical methodology to combine projections from different climate models to make meaningful conclusions, or probabilistic inferences about future ENSO. There are two main reasons for this. One is that climate models are dependent, with some models sharing components, parameterizations and code, or producing similar output^16–21^. Thus, if nine out of ten highly dependent climate models agree on a particular outcome, they could all, in fact, err; while the tenth independent model may provide the correct projection. Second, it is not clear which climate model should be trusted more. One natural assumption is that more weight should be given to models that better represent present-day ENSO^22–24^, yet no robust statistically-based method exists to measure historical ENSO performance. ENSO could be summarized using a large amount of metrics, and it is an open question how model performance in these metrics should be combined to form a single score^25^. Furthermore, both present-day model output and future projections are heavily influenced by internal climate variability^15,26–28^.

Therefore, available ENSO projections are non-probabilistic in nature. They generally offer no agreement on the magnitude or direction of future ENSO amplitude changes^8,15,29–33^. However, there are indications of likely ENSO strengthening under several forcing scenarios from the most recent generation of climate models—Climate Model Intercomparison Project phase 6 (CMIP6)^34^. Similar results are found for Eastern Pacific (EP) ENSO using earlier CMIP5 model cohort when the locations of individual ENSO variability maxima was taken into account^29^. However, there is a need to quantify the relevant outcomes probabilistically, and to expand this analysis to other ENSO metrics (e.g. skewness, seasonality, period, etc.).

**Figure 1:**
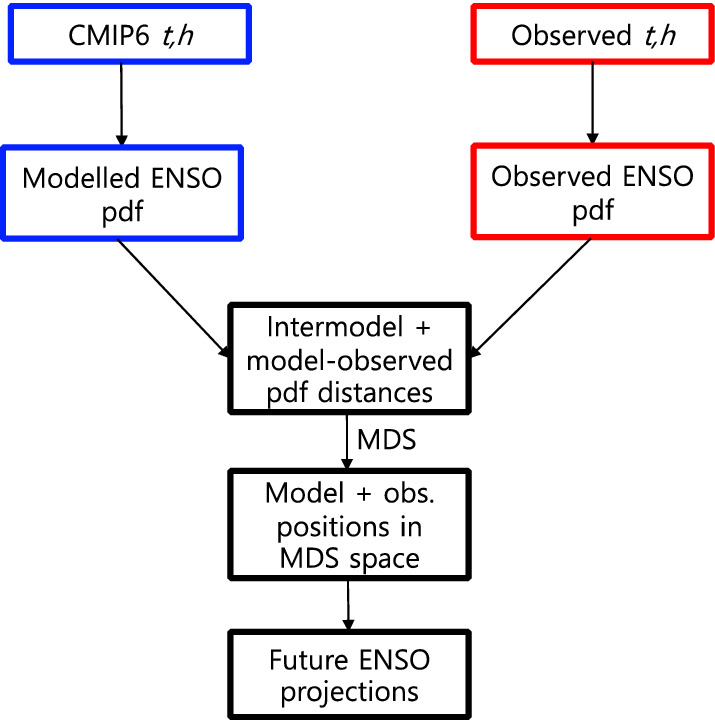
Workflow of the multi-model projection method. *t* refers to monthly Niño 3 temperatures, and *h* refers to monthly equatorial mean thermocline depths. Blue rectangles represent information derived from CMIP6 models; red rectangles represent information derived from observations. Black rectangles show information derived from the fusion of both sources of information.

**Figure 2:**
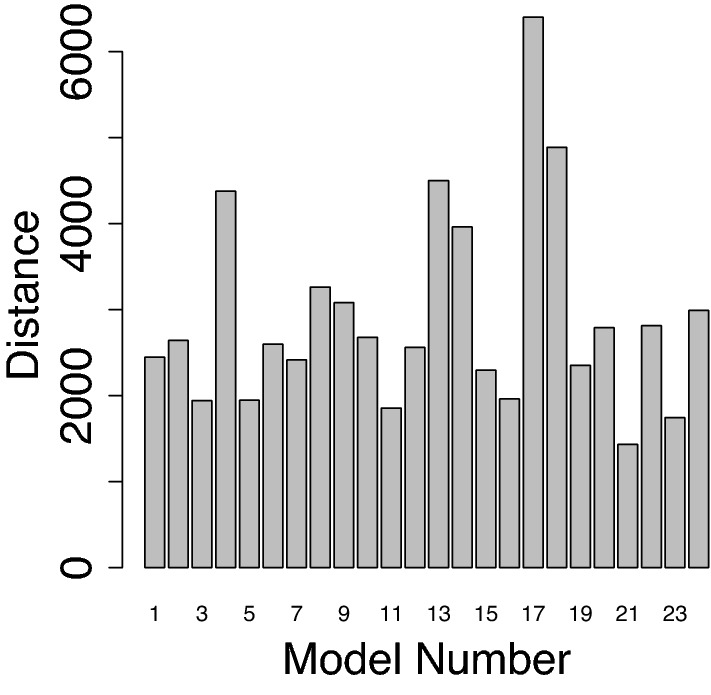
CMIP6 ENSO skill evaluation. Distances between CMIP6 models and observations, based on symmetric Kullback–Leibler divergences between 50-year PDFs of monthly ENSO paths. Model names and references can be found in Table 1.

Here, we for the first time provide probabilistic multi-model projections of ENSO that take into account both model dependence and skill at capturing the full ENSO stochastic process, assuming it can be represented by two time series variables: Niño 3 region (5°S–5°N, 150°W–90°W) SST anomalies and equatorial Pacific thermocline depth anomalies. It has been previously argued that the full information about a stochastic process is contained in the high-dimensional probability density function (PDF) of its paths, whereas other metrics (e.g. skewness, amplitude, spectrum, autocorrelation) only provide partial information^35^. We first reconstruct these PDFs covering 50 years from observations and climate models using a recently published method^35^ by fitting data-driven stochastic models (Fig. 1, “Methods”). Note that these PDFs are 600-dimensional (50 years times 12 months). The inter-model and model-observed distances are then calculated (Figs. 1, 2). We argue that these distances provide a unifying measure of models’ relative ENSO performance. These distances are then used to reconstruct model and observed positions in a low-dimensional (“configuration”) space using a method known as multidimensional scaling (MDS; Fig. 1). These positions are then input into a novel multi-model projection method that accounts for model skill and dependence^18^ to provide probabilistic projections of ENSO amplitude, skewness, seasonality, spectrum peak, kurtosis and autocorrelation changes under the shared socioeconomic pathway (SSP) forcing scenario SSP585^36^ (Fig. 1). The overall methodology for multi-model projections is not limited to ENSO and could be modified to handle other climate variables.

## Results

We first estimate 50-year PDFs using historical output of 32 CMIP6 climate models as well as observations (Table 1, “Methods”) using a recently published method^35,37^. The method can account for non-Gaussianity, long-term dependence, and cross-correlations between the SSTs and thermocline depth anomalies. We choose a subset of 24 GCMs which can be appropriately approximated by the stochastic model (Table 1).

**Table 1:** List of CMIP6 climate models used in this study.

Model name (references)	Fit OK	Model number
ACCESS-CM2^58,59^	✔	1
ACCESS-ESM1-5^60,61^		
AWI-CM-1–1-MR^62,63^		
BCC-CSM2-MR^64,65^	✔	2
CAMS-CSM1-0^66,67^	✔	3
CanESM5^68,69^	✔	4
CAS-ESM2-0^70,71^	✔	5
CESM2-WACCM^72,73^	✔	6
CIESM^74,75^	✔	7
CMCC-CM2-SR5^76,77^	✔	8
CMCC-ESM2^78,79^	✔	9
E3SM-1–1^80,81^	✔	10
EC-Earth3^82,83^		
EC-Earth3-CC^84,85^		
EC-Earth3-Veg^86,87^		
EC-Earth3-Veg-LR^88,89^	✔	11
FGOALS-f3-L^90,91^	✔	12
FGOALS-g3^92,93^	✔	13
FIO-ESM-2-0^94,95^	✔	14
GFDL-CM4^96,97^	✔	15
GFDL-ESM4^98,99^	✔	16
INM-CM4-8^100,101^	✔	17
INM-CM5-0^102,103^	✔	18
IPSL-CM6A-LR^104,105^	✔	19
KIOST-ESM^106,107^	✔	20
MPI-ESM1-2-HR^108,109^		
MPI-ESM1-2-LR^110,111^	✔	21
MRI-ESM2-0^112,113^	✔	22
NESM3^114,115^		
NorESM2-LM^116,117^	✔	23
NorESM2-MM^118,119^		
TaiESM1^120,121^	✔	24

Checkmarks indicate the climate models which are fitted by the stochastic model appropriately well. More detailed information on CMIP6 models, their components and parameterizations can be found at https://search.es-doc.org/.

We propose that a unifying measure of relative model performance with respect to observations can be a distance between these PDFs. We show model-observational distances based on symmetric Kullback–Leibler divergences in Fig. 2 (see Eqs. (4) and (5) in the “Methods” section). This method asserts that the best performing models are associated with the smallest distances. There is a large range in the model-observational distances (expressed as Kullback–Leibler divergencies), from about 1500 to more than 6000.

An important question is how the model-observational distances are related to model performance on various ENSO metrics, such as skewness, standard deviation, etc. Since there are many metrics^25^, even the best performing ENSO models can have issues at simulating some of them. On the other hand, worst ENSO models can perform well at certain metrics. The relationship between model-observational distance and a single metric is therefore expected to be weak. Furthermore, it is not immediately clear how ENSO performance at a range of metrics can be combined into a single score, that could be regressed with the distance. Thus, it is beyond the scope of this paper to quantitatively relate model distance to model metrics. Instead, we qualitatively analyze fundamental ENSO simulation issues in the five closest and five furthest models. These issues are enumerated in Table 2, and figures supporting this table are provided in the Supplementary Material.

**Table 2:** Fundamental ENSO issues for two model groups.

Model name	Main ENSO features	ENSO dynamics	ENSO noise
**Closest 5 models**			
MPI-ESM1-2-LR	No ENSO seasonality		
NorESM2-LM			
EC-Earth3-Veg-LR			
CAMS-CSM1-0			
CAS-ESM2-0		Lack of circular ENSO dynamics	Too high SST noise forcing
**Farthest 5 models**			
INM-CM4.8	Second SST amplitude peak in summer (S1). Severe underestimation of thermocline variability, almost no interannual thermocline spectral peak (S2)	No circular SST dynamics (S3)	
INM-CM5.0	Almost no interannual thermocline spectral peak (S4)	No circular SST dynamics (S5)	
FGOALS-g3			Severe underestimation of thermocline noise forcing (S6)
CanESM5	No SST seasonality (S7)		Severe underestimation of thermocline noise forcing (S8)
FIO-ESM-2–0			Severe underestimation of SST noise forcing (S9)

Fundamental ENSO issues in the five closest and five furthest CMIP6 models, based on symmetric Kullback–Leibler divergences. The references for Supplementary Figs. supporting the findings for the furthest five models are provided in brackets.

We find that the models poorly performing on the distance measure tend to be associated with more fundamental issues in simulating ENSO. For example, only three serious issues are detected in the five closest models. At the same time, we find ten issues collectively in the five furthest models. Among the poorest performing models, one common issue is the lack of noise, as quantified here by standard deviation of either monthly Niño 3 SST or equatorial thermocline depth anomaly tendencies at a specific ENSO state (e.g. Niño 3 SST and thermocline depth anomaly). Note that these standard deviations are derived not from GCM/observed output directly, but from conditional PDFs of tendencies calculated in the fitted stochastic models. Another issue found in two models (INM-CM4.8 and INM-CM5.0) is lack of circular ENSO dynamics between Niño 3 SST and equatorial thermocline depth anomalies, at least for the analyzed months. Assuming these snapshots provide a good indicator of ENSO behavior for the remaining months, this indicates that the two variables are uncoupled from each other dynamically, in contradiction to recharge oscillator theory^38–41^ and observations^37,42^. Other issues include wrong ENSO seasonality and absence of spectral peak in the thermocline anomalies. Note that some of these issues (wrong SST-thermocline dynamics) may not be reflected in simpler ENSO metrics, pointing to their drawbacks when evaluating ENSO performance.

Among the best-performing models, we found an issue with ENSO seasonality in MPI-ESM1-2-LR and too high SST noise forcing and lack of circular ENSO dynamics in the CAS-ESM2-0 model.

This qualitative analysis suggests that the distance metric is related to ENSO performance. Therefore, this metric could be used as a single measure of model performance and as a replacement for a multitude of metrics used previously. This could streamline model development, validation, and inter-comparison efforts. However, a more quantitative analysis of relationships between the distance metric and other ENSO performance metrics should be carried out in the future.

Next, we validate the methodology for the PDF reconstruction. Since the PDFs are high-dimensional it is difficult to validate their estimation in the original space. Hence, we use a method known as multi-dimensional scaling (MDS) to convert the distances into model and observed positions in a low-dimensional space. The MDS is a dimension reduction method akin to principal component analysis (PCA). Unlike the PCA, the input for MDS is not modelled values, but inter-model distances^43^. This procedure allows for better visualization of the PDFs. The output for the MDS are positions of the models in a low dimensional space of principal coordinates R_1_, … R_p_. The MDS suggests that the uncertainties associated with estimation of the observed and modelled PDFs is reasonably low for the three leading MDS dimensions (Supplementary Note).

**Figure 3:**
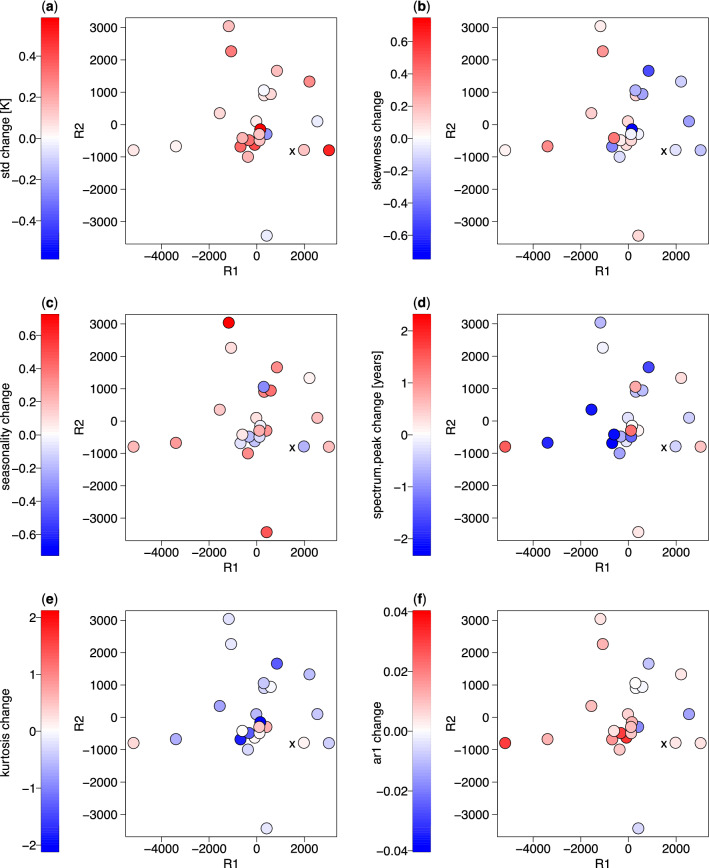
Results of multi-dimensional scaling (MDS) analysis of ENSO probability density functions from CMIP6 climate models. Changes between periods 1850–1949 to 2040–2099 as a function of MDS principal coordinates for Niño 3 (**a**) standard deviation, (**b**) skewness, (**c**) seasonality, (**d**) ENSO spectrum peak, (**e**) kurtosis and (**f**) autocorrelation from CMIP6 models (color) under the SSP585 forcing scenario. Observed coordinates are denoted by black crosses. R_1_ and R_2_ are the first two principal coordinates of the observed and modelled PDFs of ENSO paths.

The multi-model projection method uses a low-dimensional representation of modelled positions^18^. To reconstruct those positions, the MDS was performed using the inter-model and model-observational distances from the first ensemble members (“r1i1p1f1”) for the 24 models and observations. Five and six (*e* = 5, 6) dimensional representations are chosen as they yield the best performance (see “Methods”). To illustrate relationships between MDS coordinates and future changes, the response plots of 1850–1949 to 2040–2099 changes in six ENSO properties over the Niño 3 region (standard deviation, skewness, seasonality, ENSO spectrum peak, kurtosis and lag-1 autocorrelation) are shown in Fig. 3 as a function of the first two principal MDS coordinates, R_1_ and R_2_. We note that these coordinates have no physical meaning, although they could be in theory correlated with certain physical quantities. There is a degree of randomness in the CMIP6 model responses due to internal variability and due to the uncertainty in the estimation of the MDS coordinates. However, some marked clusters are visible. They include the cluster of intensified, more persistent ENSO with lower kurtosis and shorter spectral peak in the middle-left of the configuration space (Fig. 3a,d,e,f), and decreased skewness at high R_1_ values vs. increased skewness as low R_1_ values (Fig. 3b).

We then apply the recently developed multi-model projection method that accounts for model skills and dependence^18^. The key idea of the method is relaxing the assumption commonly used in the popular multi-model projection method called “Bayesian model averaging”^23,44^. That method constructs future projections as the weighted average of modelled PDFs. The weight is usually associated with model skill at representing present-day climate. This assumption is that in reality only one model can be correct, which does not properly represent model dependence. We relax this assumption. However, this leads to more complex mathematical expressions that can no longer be easily evaluated. Nonetheless, if we formulate model correctness hypotheses as regions in parameter space we can obtain future PDFs by instead sampling from the subspace of the parameter space where at least one model is correct^18^.

The method, modified from its original version to fit the problem at hand, states that associated with each future change Δ (value in the future period minus the value in the historical period) there is a correctness indicator for each climate model *i*, depending on its relative position in space of R_1_, …, R_e_, and future change (${r}_{1,{m}_{i}},{\dots , r}_{e,{m}_{i}}, {\Delta }_{i})$, and the position of “truth” consisting of observations and given future change (*r*_1,o_,…, *r*_e,o_, Δ). The tolerance controlling the model correctness cutoff is controlled by a parameter *k* (see “Methods”). The method works by sampling all pairs of (*k*, Δ) for which at least one model is considered correct (see “Methods”). The prior probability of *k* is determined using cross-validation (“Methods”). It has been mathematically proven that this methodology considers both model skill at capturing present-day observations, and all orders of model dependence (e.g. dependence of each model on other models individually, on pairs of other models, triplets of other models, and so on) both in terms of present-day output and future projections^18^. We address the uncertainty in the correct MDS dimensionality by simply combining the projections from simulations using e = 5 and e = 6.

We note that model dependence and model non-exclusivity are related terms. Here, we mathematically define dependence in terms of model non-exclusivity as $\mathrm{\Finv }=\sum_{1\le i<j\le l}p({M}_{i}\cap {M}_{j})$ where $p({M}_{i})$ is the probability that model *i* is correct, and *l* is the number of available models. A measure of non-exclusivity (or dependence) for a particular model could be similarly defined as ${\mathrm{\Finv }}_{i}=\sum_{1<j<l,j\ne i}p({M}_{i}\cap {M}_{j})$. In other words, it is a tendency for a model to be jointly correct with other models, and for its output to cluster together with other models.

**Figure 4:**
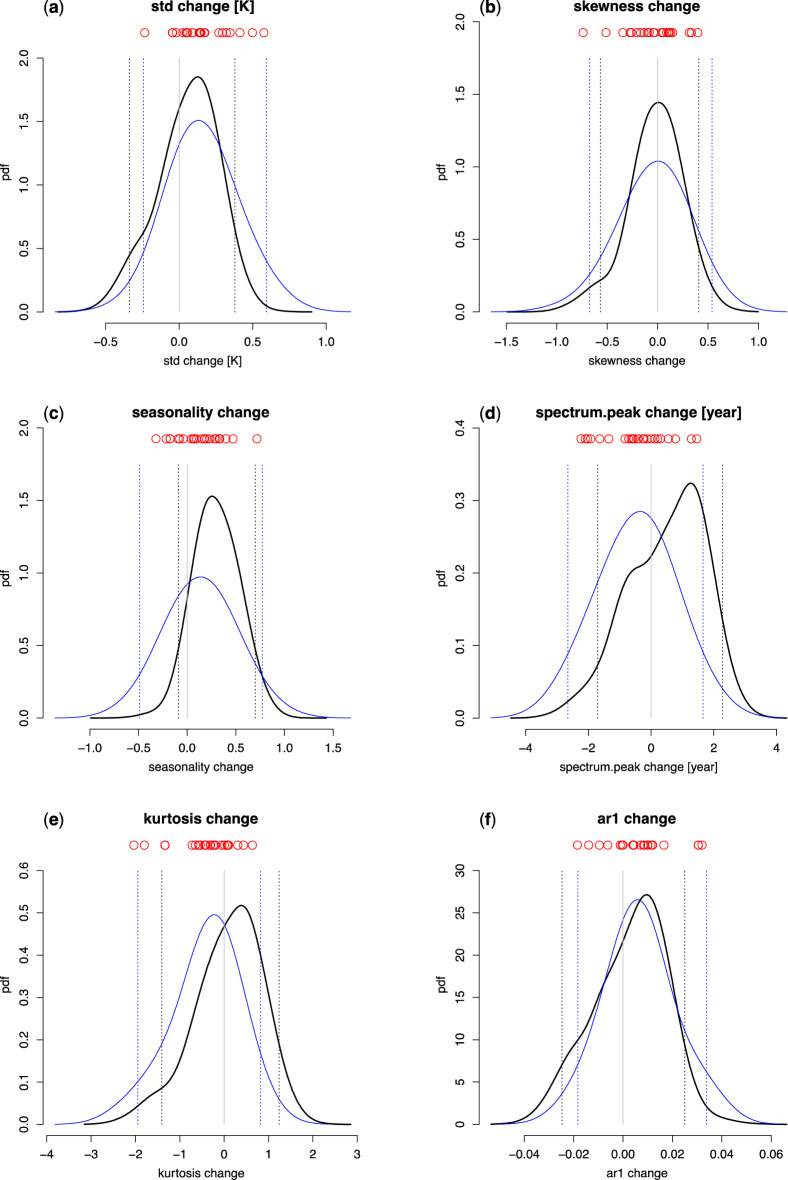
ENSO probabilistic projections from CMIP6 climate models. Probabilistic multi-model projections for changes in Niño 3 (**a**) standard deviation, (**b**) skewness, (**c**) seasonality, (**d**) ENSO spectrum peak, (**e**) kurtosis and (**f**) lag-1 autocorrelation between years 1850–1949 and 2040–2099 from CMIP6 climate models under the SSP585 emissions scenario (black lines). The projections are compared to resampling of PDFs obtained using kernel density estimation from actual modelled changes (thin blue lines). Dotted vertical lines represent respective 90% confidence intervals. Vertical grey lines are zero lines. Individual model projections are shown by red circles.

We show the future projections for key ENSO properties in Fig. 4, while the summary of the results is presented in Table 3. The projection results are compared with simple poor-man’s resampling of PDFs construed from 24 available future modelled changes using kernel density estimation (KDE; see “Methods”). We note that the KDE-based poor man’s approach is statistically wrong, as it incorrectly assumes that modelled changes are independent samples from the underlying PDF. In addition, it does not account for differential model skill at simulating ENSO. Thus, the poor man’s projections are shown for mere comparison with the novel method.

**Table 3:** Summary of ENSO multi-model projections.

Variable	Projection mean	Probability of an increase (novel method)	Probability of an increase (KDE resampling)	90% confidence interval reduction
Standard deviation [K]	0.058	64%	**73% (likely)**	14%
Skewness	− 0.026	49%	47%	20%
Seasonality	0.30	**89% (likely)**	64%	37%
ENSO spectrum peak [year]	0.5	**67% (likely)**	37%	7.7%
Kurtosis	0.092	58%	**32% (unlikely)**	4.5%
Lag-1 autocorrelation	0.0029	61%	**67% (likely)**	4.5%

Key properties of multi-model probabilistic ENSO projections for changes between years 1850–1949 and 2040–2099 from CMIP6 climate models under the SSP585 forcing scenario using the novel method and compared to the poor man’s approach of resampling of PDFs obtained using kernel density estimation. Probability of increase is re-stated using the IPCC uncertainty guidance note terminology^45^**.**

Likely changes (using the IPCC guidance note terminology) are shown in bold.

Our work projects a 64% chance of ENSO amplification (about as likely as not in the language of the IPCC uncertainty guidance note^45^), with the mean strengthening of just 0.058 K (Fig. 4a, Table 3). The likelihood of an increase is down from 73% (likely) obtained from a simple resampling of the KDE-based PDF. Earlier studies based on Climate Model Intercomparison Project phase 5 (CMIP5) yielded inconclusive results: while one work projected future ENSO amplification^29^, other studies did not agree on the direction of future ENSO change^8,15,30^. Furthermore, a centennial time-scale modelling study argued that any possible intensification under increased atmospheric CO_2_ concentrations would be temporary, with weaker ENSO on the long term^31^.

Yet, the newest CMIP6 climate model ensemble shows robust increases in ENSO amplitude^34^, with 88.4% percent of models predicting an increase in the twenty-first century over the twentieth century under the SSP585 forcing scenario. The resampled KDE-based PDF from our work shows a smaller 73% probability of an increase, and the difference between the studies could be due to a different region (we use Niño 3 compared to Niño 3.4 used in that work), and period. However, when model skill and dependence are considered, the percentage of future increases falls even more down to 64%. Indeed, out of three most likely models based on our analysis, one (CAM-ESM2-0) projects future ENSO weakening of -0.044 K.

Large uncertainty remains regarding future skewness projections (49% probability of an increase; Table 3, Fig. 4b). A similar percentage (47%) is obtained using the poor man’s PDF resampling (Table 3). Previous work using CMIP5 model generation suggested that future skewness may decrease^15,30^. The gap between our and previous works could be due to a number factors, including using different sets of global climate models, periods for calculating the changes, and regions for averaging ENSO anomalies.

Our approach projects a robust increase in ENSO seasonality (89% chance), with a mean increase of 0.3 (Fig. 4c, Table 3). We note that observed seasonality (defined here as the ratio of ENSO standard deviation for the most variable month over one for the least variable month) for years 1915–2016 is 1.9. Therefore, the projected mean increase is 16%. These results are markedly different from the KDE-based “poor-man’s” projections, which indicate only a 64% chance of an increase. Three most likely models under our analysis, MPI-ESM1-2-LR, CIESM and CAS-ESM2-0, project increases of 0.23, 0.33 and 0.47, respectively. These results are in contrast to inconclusive findings previously reported based on a set of CMIP5 climate models^15^.

Furthermore, our work indicates a likely (67% probability) lengthening of ENSO period. This is in stark contrast to a 37% chance of lengthening found in the KDE-based projection PDF. Furthermore, both lower and upper endpoints of the 90% confidence intervals are shifted towards higher values, with a small (7.7%) decrease in uncertainty (Fig. 4d, Table 3). The most likely outcome (mode of the PDF) is that of 1.3 years lengthening of ENSO period, which mirrors the value output from the highest-probability model, MPI-ESM1-2-LR. However, the model with the second highest probability, CIESM, showcases a small decrease of -0.55 years, which may explain a bump in the projection PDF around that value (Fig. 4d). ENSO period has not been previously intensely scrutinized, and available work did not project any large future changes in the ENSO spectrum^46^.

Furthermore, our projections offer mixed results on future kurtosis changes (58% chance of an increase). These results are markedly different from the likely (68% chance) decreases found using the poor man’s approach of resampling the KDE-based PDF. For this variable, there is only a minor reduction (4.5%) of uncertainty owing to considering model skill and dependence. There is additionally a 61% chance of increased month-on-month autocorrelation, a small drop down from 67% (likely) under a poor-man’s approach. Again, there is only a small (4.5%) decrease in projection uncertainty compared to the poor-man’s approach in case of autocorrelation.

Compared to the poor-man’s approach of resampling PDFs of future changes obtained through the KDE method, there is a reduction in uncertainty for all projection variables. The smallest reduction (4.5%) is found for kurtosis and lag-1 autocorrelation, while the largest reduction (37%) occurs for seasonality projections. The reduced projection uncertainty is thus a major benefit of our approach. We remind the reader that the poor-man’s approach should never be used to make probabilistic projections; we include it solely with the purpose of showcasing the reduction of uncertainty owing to the novel projection method.

An advantage of our method compared to deterministic approaches is that it allows us to characterize the range of uncertainty using various metrics, for example, the 90% confidence interval. Reflecting the overall future projected increase in seasonality, the associated 90% confidence interval is asymmetric around zero, ranging from − 0.089 to 0.70 (Fig. 4c). A spectrum peak shortening or lengthening by two years cannot be ruled out (Fig. 4d). Large decreases in kurtosis are more likely than increases of similar magnitude due to a large lower tail (Fig. 4e). In addition, we note that the model range is not always a reliable indicator of the projection uncertainty range. For example, there are incidences where at least one of the bounds of the 90% confidence interval is outside of the model range (Fig. 4). This illustrates the limits of using model range as an indicator of projection uncertainty.

Furthermore, we find that properly accounting for model dependence is numerically important. An existing popular method of combining models known as “Bayesian model averaging (BMA)” handles dependence using the following assumption: only one model can be true at a time (“mutually exclusive” events). In other words, if one model is correct, then all other models must be incorrect: $p\left({M}_{i}|{M}_{j}=0\right),i\ne j$. If this is the case, then the sum of model probabilities is exactly one within our sample space (which asserts that at least one climate model is correct): $\sum_{i=1}^{l}p\left({M}_{i}\right)=1$, where *l* is the number of models. More generally, the Law of Total Probability states that:

1 $$p\left(\bigcup_{i=1}^{l}{M}_{i}\right)=1=\sum_{i=1}^{l}p\left({M}_{i}\right)-\sum_{1\le i<j\le l}p\left({M}_{i}\cap {M}_{j}\right)+\sum_{1\le i<j<k\le l}p\left({M}_{i}\cap {M}_{j}\cap {M}_{k}\right)-\cdots +{(-1)}^{l-1}p\left({M}_{1}\cap {M}_{2}\cap \cdots {\cap M}_{l}\right),$$

with the first term $\sum_{i=1}^{l}p({M}_{i})$ being greater than one. Previous work hypothesized that the assumption of model exclusivity in the BMA (e.g. ignoring probabilities of model pairs $\sum_{1\le i<j\le l}p\left({M}_{i}\cap {M}_{j}\right)$, triplets $\sum_{1\le i<j<k\le l}p\left({M}_{i}\cap {M}_{j}\cap {M}_{k}\right)$, etc.) may be relatively unimportant when the dimensionality of observational constrains on climate models is high (> 2). However, here we find that even with five MDS dimensions for case of standard deviation projections, $\sum_{i=1}^{l}p\left({M}_{i}\right)=2.11\ne 1$. This suggests that accounting for model dependence in a proper way is critical even when multiple dimensions are used to constrain climate models.

Our work is subject to several caveats. First, we only consider time series aspects of ENSO without any spatial information and constrain our analysis to the Niño 3 region. We choose this region as it is important for dynamical ENSO evolution within the recharge oscillator theory^39–41,47^. However, previous work has found that the ENSO projections differ when they are made at the locations of model-dependent centers of ENSO variability^29^. Considering other ENSO regions is subject of future work. Second, we neglect the uncertainty in the present-day MDS location of observations. However, sensitivity analysis to the observations (see “Methods” and Supplementary Note) suggests that the errors in the observational estimate in three leading MDS dimensions are small. In addition, MDS location of pseudo-observations and the observations are similar, suggesting that the errors introduced due to fitting the stochastic model are small. Third, we assume that tolerance for defining model credibility regions is the same for all models. A more thorough treatment of model uncertainties should be done in the follow-up work. Fourth, we assume that ENSO over the periods used for the analysis is stationary, which has been suggested by a previous study^48^. Fifth, there is a discrepancy in the observational and the modelled periods: the GCMs output is used over the period 1871–2014, while the observations cover the period from 1915 to 2016. The length of the observational period is limited by the presence of a dense observational network; we found that extending the period back in time leads to the rapid deterioration of the stochastic model fits. At the same time, discarding earlier GCM data is expected to increase the errors in the estimation of GCM positions in the configuration space. However, our analysis suggests that the length of model time series is sufficient, and there are no indications internal variability is an issue. This is supported by proximity of different ensemble members of same GCMs in leading MDS space dimensions (Supplementary Note).

## Conclusions

Here we for the first time present multi-model probabilistic projections of ENSO properties. The projections account for the differential skill of climate models at capturing present-day high-dimensional PDF of ENSO paths (this PDF completely describes the underlying stochastic process assuming it is limited by 50 years), and for the dependence of climate models. We find that future ENSO will likely display more seasonality (89% probability) and have a longer spectrum peak (67% probability), with the most likely spectrum peak lengthening of 1.3 years. We compare our projections with a poor-man’s approach or sampling the probability density of future changes obtained through kernel density estimation. We stress that such poor man’s approach should not be performed in practice as it does not account for model skill and dependence, and we include it here only for comparison purposes. We find that the novel approach reduces the projection uncertainty (measured by the width of 90% confidence intervals) by 4.5—37%, depending on the projection variable, with the largest reduction for ENSO seasonality. In addition, projections in some cases change qualitatively compared to the poor man’s approach (based on the IPCC terminology). For example, kurtosis increases change from “unlikely” to “about as likely as not”, and lag-1 autocorrelation increases change from “likely” to “about as likely as not”. Furthermore, we attest that accounting for model dependence is important numerically, even when observational constraints include several dimensions.

In addition, we propose a unifying PDF-based metric of climate model performance at ENSO time series properties and rank the ENSO performance of CMIP6 models. Future work should extend this method to consider the spatial structure of ENSO. Our method is not restricted to ENSO or atmospheric sciences and can be used with other monthly cyclostationary time series.

## Methods

### Data

We use 32 climate models from the CMIP6 project. The list of models is given in Table 1. These models were chosen as only they have bug-free output necessary to perform this analysis. Historical run ensemble members r1i1p1f1 for years 1871–2014 are used for fitting the stochastic models, while historical output for years 1850–1949 and future output under the SSP585 forcing for years 2040–2099 is used to calculate future changes. In addition, ensemble members r2i1p1f1 for three GCMs (BCC-CSM2-MR, CAMS-CSM1-0 and INM-CM5-0) are also utilized for years 1871–2014. All relevant monthly output is horizontally interpolated onto a 1°x1° grid using nearest neighbor interpolation. To calculate Niño 3 index *t*, we first perform the spatial average of SST over the Niño 3 region (5°S–5°N, 150°W–90°W), then calculate anomalies from monthly means, and then linearly detrend these anomalies over each respective period.

A similar procedure is used for calculating equatorial mean thermocline *h* over the Pacific for the latitudes 5°S–5°N. First, for each interpolated grid point the depth of the 17-degree isotherm is obtained from monthly potential temperature fields. Preliminary data analysis indicates that the difference between potential temperature and temperature can be ignored for the thermocline depth calculations. Then, the depth is horizontally averaged over the equatorial Pacific, the annual cycle is removed, and the anomalies are linearly detrended.

The data extraction procedure for observations (years 1915–2016) is similar, except no interpolation of the observed datasets is done. Observed SST anomalies originate from the ERSSTv5 dataset^49^. The observational sources used to calculate thermocline depth anomalies include SODAv2.2.4 reanalysis for years 1915–2010^48^, and GODAS reanalysis for years 2011–2016^50^. Unlike the GCMs, thermocline depth from the SODAv2.2.4 reanalysis is obtained from in-situ temperature fields.

We also perform sensitivity analysis with respect to the observations. To do that, we construct the second observational dataset, where SST anomalies originate from the HadISST dataset^51^**,** and the sources for ocean temperature used to calculate the thermocline depth anomalies are split between two datasets. Specifically, they come from SODAv2.2.4 for years 1915–1957 and from ORAS4 for years 1958–2016^52^**.** Note that to our knowledge SODAv2.2.4 is the only available ocean reanalysis available prior to year 1958.

### Stochastic models

Stochastic models are fitted to the 1871–2014 monthly Niño 3 index and thermocline depth following previous work^35,37^. Specifically, the conditional probabilities of monthly Niño 3 changes ($\Delta t$) and thermocline depth changes ($\Delta h$) given the state (*t*,*h*) are found from the joint probabilities $p(\Delta t,t,h)$ and $p(\Delta h,t,h)$, which are, in turn, estimated using kernel density methods separately for each calendar month^37^. The non-Markov part of the model describes the normal transforms of the SST and thermocline depth changes using the multivariate normal distribution. Specifically,

2 $${\xi }_{t}^{(j)}={\Phi }^{-1}\left(F\left(\Delta {t}^{\left(j\right)}|{t}^{\left(j\right)},{h}^{\left(j\right)},{m}^{\left(j\right)}\right)\right),$$

where subscript *t* stands for temperature, superscript indicates time index, ${\Phi }^{-1}$ is the inverse standard normal cumulative distribution function (CDF), *F* is conditional cumulative distribution function (CCDF), and *m* is month. In Eq. (2) the changes are defined as ${\Delta t}^{(s)}={t}^{(s+1)}-{t}^{(s)}$ and similarly for the thermocline depth anomalies. A similar equation is used for normal transforms of thermocline depth changes ${\xi }_{h}^{(j)}$. The transforms are then collected into a single vector which is modelled using the multivariate normal distribution: ${\varvec{\xi}}=[{\xi }_{t}^{\left(s\right)},{\xi }_{h}^{\left(s\right)},{\xi }_{t}^{\left(s+1\right)},{\xi }_{h}^{\left(s+1\right)},\dots ,{\xi }_{t}^{\left(s+v\right)},{\xi }_{h}^{\left(s+v\right)}]\sim N(0,{\varvec{\Sigma}})$. The covariance matrix ${\varvec{\Sigma}}$ of this distribution is estimated from data. The model allows to find the theoretical cyclostationary probability of any sequence of ENSO trajectories **z**:

3 $$p\left(\mathbf{z}\right)=p\left({t}^{\left(s\right)},{h}^{\left(s\right)},\dots ,{t}^{\left(s+v+1\right)},{h}^{\left(s+v+1\right)}\right).$$

The stochastic models are fitted using a procedure that has been modified from previous work^35,37^. Consistent with these studies, we use bandwidth $\mathrm{S}\mathbf{K}$ during the kernel density estimation, where S is a numeric variable and $\mathbf{K}$ is the two-stage plug-in estimator for the bandwidth matrix^53^. However, the procedure for estimating S is different. Here, we develop a systematic procedure for choosing S. First, for each GCM, different values of S are tried, starting from an initial value of 2, and decreasing in steps of 0.25. For each value, the Markov part of the model is first fitted on 50×50×50 grids of ($\Delta t,t,h)$ and ($\Delta h,t,h)$. For the non-Markov part, we set the size of ${\varvec{\Sigma}}$ to cover the period of 143 years (1 year less than the length of the model output), and optimize ${\varvec{\Sigma}}$ following previous work^35^. For each S, we keep track of the AIC of the model output over years 1871–2013. If the AIC at the next value of S in the sequence starts increasing, or if a multimodality is detected in any of the marginal PDFs $p(\Delta t)$, $p(t)$ or $p(h)$ obtained from the joint PDF $p(\Delta t,t,h)$ for the representative month of January, we keep the current value of S as the final value. Afterwards, we obtain the multi-model mean of S determined in this way across all GCMs (0.98) and re-fit the stochastic models for all 32 GCMs using this value. We have tried using different S values for each GCM, however, it led to inconsistent PDF estimation based on the MDS analysis. Therefore, we opt to use the same value for all GCMs. This value of S is also used for fitting the ensemble member r2i1p1f1 output for three GCMs.

We assess S separately for the observations in a similar way over the period 1915–2016. In this case, ${\varvec{\Sigma}}$ covers 101 years, and AIC is calculated for years 1915–2015. We estimate the observed S to be 1.5. It is natural to hypothesize that S is different between the GCMs and the observations because of the different length of the data. Specifically, when the sample size is smaller, we need to make sure that there is enough smoothing to remove spurious multimodalities and discontinuities in the PDFs, hence we expect a larger S.

The goodness of fit of the stochastic models is estimated using their relative error at representing the original GCM output and comparing this error to the relative error of long runs of the stochastic model fitted to EC-Earth3-Veg GCM at representing its own 144-year time segment. EC-Earth3-Veg is chosen due to its positive performance on a number of ENSO-related metrics^25^. The procedure is described in previous work^35^, except the length of the short segment to which the long runs are compared to is different. The stochastic model is deemed to fit reasonably well to 24 out of 32 models (Table 1). We thus choose these models for further analysis.

### Inter-model and model-observational distances

Once the model and observed monthly ENSO path PDFs $p\left(\mathbf{z}\right)$ for 50-year periods are obtained, we set out to calculate distances between 600-dimensional PDFs for all GCMs, and observations. Here we use the symmetric version of the Kullback–Leibler divergence:

4 $${{\gamma }_{ij}=D}_{k}\left({p}_{{M}_{i}}\left(\mathbf{z}\right),{q}_{{M}_{j}}\left(\mathbf{z}\right)\right)={D}_{k}\left({p}_{{M}_{i}}\left(\mathbf{z}\right)||{q}_{{M}_{j}}\left(\mathbf{z}\right)\right)+{D}_{k}\left({q}_{{M}_{j}}\left(\mathbf{z}\right)||{p}_{{M}_{i}}\left(\mathbf{z}\right)\right),$$

where

5 $${D}_{k}\left({p}_{{M}_{i}}\left(\mathbf{z}\right)||{q}_{{M}_{j}}\left(\mathbf{z}\right)\right)={\int }_{\mathbf{z}}{p}_{{M}_{i}}\left(\mathbf{z}\right)\mathrm{ln}\frac{{p}_{{M}_{i}}\left(\mathbf{z}\right)}{{q}_{{M}_{j}}\left(\mathbf{z}\right)}d\mathbf{z}$$

is the standard Kullback–Leibler divergence. Here ${p}_{{M}_{i}}\left(\mathbf{z}\right)$ and ${q}_{{M}_{j}}\left(\mathbf{z}\right)$ represent PDFs of vector $\mathbf{z}$ being simulated by the *i*th and *j*th models, respectively. Note that this is a multidimensional integral over 50-year trajectories of Niño 3 SST and equatorial thermocline depth. Symmetric Kullback–Leibler divergences have been previously used in the context of dimensionality reduction of PDFs^54^. This integral is evaluated using Monte Carlo integration. Specifically, for each pair of GCMs or observations samples from ${p}_{{M}_{i}}\left(\mathbf{z}\right)$ are generated using direct simulation of the stochastic model, and then ${D}_{k}\left({p}_{{M}_{i}}\left(\mathbf{z}\right)||{q}_{{M}_{j}}\left(\mathbf{z}\right)\right)$ is obtained by taking a simple mean of the fraction term $\mathrm{ln}\frac{{p}_{{M}_{i}}\left(\mathbf{z}\right)}{{q}_{{M}_{j}}\left(\mathbf{z}\right)}$ over the random **z** samples, and ${D}_{k}\left({q}_{{M}_{j}}\left(\mathbf{z}\right)||{p}_{{M}_{i}}\left(\mathbf{z}\right)\right)$ is calculated in the similar way. We use 1000 Monte Carlo samples. Preliminary data analysis shows that this number of samples is sufficient to estimate the symmetric Kullback–Leibler divergence with reasonable accuracy.

### Multidimensional scaling (MDS)

Multidimensional scaling takes in inter-model (or model-observational) distances, and finds model locations in the space of given dimensionality^55,56^. More specifically, the input to the method is the $n\times n$ matrix of inter-model distances ${{\varvec{\Gamma}}=(\gamma }_{ij})$. These distances are first converted to the $n\times n$ matrix ${\mathbf{A}=(a}_{ij})$, where ${a}_{ij}=-{\frac{1}{2}\gamma }_{ij}^{2}$. Next, the symmetric $n\times n$ matrix $\mathbf{B}=\mathbf{H}\mathbf{A}\mathbf{H}$ is formed, where $\mathbf{H}={\mathbf{I}}_{{\varvec{n}}}-{n}^{-1}{\mathbf{J}}_{{\varvec{n}}}$ and ${\mathbf{J}}_{{\varvec{n}}}$ is an $n\times n$ matrix of ones. The matrix $\mathbf{B}$ is then spectrally decomposed to find eigenvalues and eigenvectors: $\mathbf{B}=\mathbf{V}{\varvec{\Lambda}}{\mathbf{V}}^{{\varvec{T}}}$, where ${\varvec{\Lambda}}=\mathrm{diag}({\lambda }_{1},\dots {,\lambda }_{n})$ is the diagonal matrix of the eigenvalues of $\mathbf{B}$ sorted in non-increasing order and $\mathbf{V}=[{\mathbf{v}}_{1},\dots {,\mathbf{v}}_{{\varvec{n}}}]$ is the matrix whose columns are the eigenvectors of **B**. Suppose that the required dimension of analysis is $e$, and that $e<{e}^{^{\prime}}$, where ${e}^{^{\prime}}$ is the number of positive eigenvalues of **B**. We define ${{\varvec{\Lambda}}}_{1}=\mathrm{diag}({\lambda }_{1},\dots {,\lambda }_{e})$ to be the matrix of $e$ largest eigenvalues of **B** in non-increasing order, and ${\mathbf{V}}_{1}$ to be the associated matrix of $e$ eigenvectors. Then,

6 $$\mathbf{B}={\mathbf{V}}_{1}{{\varvec{\Lambda}}}_{1}{\mathbf{V}}_{1}^{{\varvec{T}}}=\mathbf{R}{\mathbf{R}}^{{\varvec{T}}},$$

where $\mathbf{R}={\mathbf{V}}_{1}{{\varvec{\Lambda}}}_{1}^{1/2}=\left[\sqrt{{\lambda }_{1}}{\mathbf{v}}_{1},\dots ,\sqrt{{\lambda }_{e}}{\mathbf{v}}_{{\varvec{e}}}\right]=[{\mathbf{r}}_{1},\dots ,{\mathbf{r}}_{\mathbf{n}}{]}^{T}$, and ${\mathbf{r}}_{1},\dots {\mathbf{r}}_{\mathbf{n}}$ are the principal coordinates of the observational and model locations in the $e$-dimensional space. The inter-model and model-observational distances ${d}_{ij}$ in the reduced-dimensional space closely approximate the original distances ${\gamma }_{ij}$.

Here, we select the MDS dimension $e$ using cross-validation by excluding each model one at-a-time, and comparing multi-model projections (see “Cross-Validation” subsection) to the excluded model outputs. We achieve the best mean absolute error of the mean for *e* = 5, and best mean absolute error of the mode for *e* = 6. We therefore combine results for these dimensionalities when performing future projections (subsections below). We perform MDS twice: first with observations, canonical 24 models, additional runs of three GCMs, pseudo-observations, and second set of alternative observations (Supplementary Note). The purpose of this analysis is to illustrate the uncertainties involves in the estimation of the MDS coordinates and the original PDFs. The second time we perform the MDS is for the purpose of multi-metric future projections. Here, we use only the observations and the canonical r1i1p1f1 runs of the 24 GCMs.

When performing the MDS the first row of the distance matrix always involves the default observational set, while the rest of the rows involve climate models, pseudo-observations or additional observations.

### Future multi-model projections

We define hypotheses about climate model correctness by modifying previous work^18^ as regions in parameter space. Consider that each *i*th model is associated with the coordinate ${{\varvec{\theta}}}_{{\varvec{i}}}=\left({r}_{1,{m}_{i}},\dots {,r}_{e,{m}_{i}}, {\Delta }_{{m}_{i}}\right)$ in the space of $e$ MDS coordinates and future changes $\Delta .$ Note that here changes represent long-term changes between the historical (years 1850–1949) and the future period (years 2040–2099). These coordinates can be normalized to have the inter-model range of one in each dimension; let the normalized coordinates be:

7 $${{\varvec{\theta}}}_{{\varvec{i}}}^{\boldsymbol{*}}=\left({r}_{1,{m}_{i}}^{*},\dots ,{r}_{e,{m}_{i}}^{*},{\Delta }_{{m}_{i}}^{*}\right)=\left(\frac{{r}_{1,{m}_{i}}}{{G}_{1}},\dots ,\frac{{r}_{e,{m}_{i}}}{{G}_{e}},\frac{{\Delta }_{{m}_{i}}}{{G}_{\Delta }}\right),$$

where $${G}_{j}$$ is the range of model coordinates in the dimension *j*, and $${G}_{\Delta }$$ is the range of future modelled changes. Each model *i* is considered to be correct ($${H}_{i})$$ under a given “true” future climate change $$\Delta$$ if the distance between its normalized coordinates $${{\varvec{\theta}}}_{{\varvec{i}}}^{\boldsymbol{*}}$$ and the normalized coordinates of the truth (consisting of the normalized MDS positions of observations and the normalized “true” future climate change $${\Delta }^{*})$$ $${{\varvec{\theta}}}^{\boldsymbol{*}}=\left({r}_{1,o}^{*},\dots ,{r}_{e,o}^{*},{\Delta }^{*}\right)=\left(\frac{{r}_{1,o}}{{G}_{1}},\dots ,\frac{{r}_{e,o}}{{G}_{e}},\frac{\Delta }{{G}_{\Delta }}\right)$$ is within a given value *k*:

8 $${\varvec{D}}\left({{\varvec{\theta}}}_{{\varvec{i}}}^{\boldsymbol{*}},{{\varvec{\theta}}}^{\boldsymbol{*}}\right)=\sqrt{{\left(\frac{{r}_{1,{m}_{i}}-{r}_{1,o}}{{G}_{1}}\right)}^{2}+\cdots +{\left(\frac{{r}_{e,{m}_{i}}-{r}_{e,o}}{{G}_{e}}\right)}^{2}+{\left(\frac{{\Delta }_{{m}_{i}}-\Delta }{{G}_{\Delta }}\right)}^{2}}<k.$$

The difference between this formulation and the one previously employed^18^ is that the current formulation uses hyperspheres in an *e*-dimensional space as model correctness regions, whereas the previous formulation uses hypercubes. The hypersphere formulation appears to be more intuitive to us, since it, unlike the hypercube formulation, could be represented using distances. In addition, its region boundaries are smooth, unlike those of the hypercube formulation.

Furthermore, we define sample space $\Omega$ as the region in space ($\Delta ,k)$ where at least one climate model is correct: ${H}_{1}\cup \cdots {\cup H}_{l}$, where *l* = *n*-1 is the number of models. We choose the prior for *k* to be a half-normal distribution $k\sim {N}^{+}(0,{\sigma }^{2})$ following prior work^18^. Here, $\sigma$ is the parameter chosen by the user. We find an appropriate value for it using cross-validation (described below). Future projections are made using the marginalization theorem:

9 $$p\left(\Delta \right)=\int p\left(\Delta ,k\right)dk=\int p\left(\Delta |k\right)p\left(k\right)dk\propto \int {1}_{\Omega }p\left(k\right)dk,$$

where ${1}_{\Omega }$ is an indicator function for membership in the set $\Omega$ (it is one over the set $\Omega$, and 0 outside of this set). Samples from ${1}_{\Omega }p\left(k\right)$ are taken using rejection sampling. We perform future projections using runs r1i1p1f1 from the 24 selected models.

We address the uncertainty in the number of dimensions *e* by combining projections from *e* = 5 and 6, as these were the best-performing dimensionalities during cross-validation (see subsection below).

### Cross-validation

We use cross-validation to optimize the number of MDS dimensions, as well as the parameter $\sigma$. We try MDS dimensionalities *e* ranging from 2 to 8. For each dimensionality, each model is excluded from the analysis one-by-one (except the model CAS-ESM2-0, which is an outlier in the MDS space), multi-model projections of Niño 3 standard deviation changes for years 1850–1949 to 2040–2099 are performed using the remaining 23 models and compared with the modelled change in the excluded model. Then, an appropriate $\sigma$ is obtained such that it provides well-calibrated confidence intervals for the standard deviation changes (the 90% confidence intervals having an empirical coverage of around 90%). 50,000 samples from the joint PDF are used. An additional experiment in 5 MDS dimensions with a different initial random seed indicates that 50,000 samples are enough to reasonably approximate the marginal PDF of future projections. We analyze the mean absolute error of the mean and mean absolute error of the mode for different dimensions. We find that the mean absolute error of the mean is smallest for *e* = 5, while the mean absolute error of the mode – for *e* = 6. Without any further information on the likelihood of different *e* values, we simply combine projections from both dimensions (which is effectively Bayesian model averaging with an equal probability for two competing models with different values of the hyperparameter *e*).

A sensitivity cross-validation experiment for *e* = 5 where all MDS dimensions have been equally scaled using the mean range ${G}^{^{\prime}}=\frac{1}{5}\sum_{j=1}^{5}{G}_{j}$ has resulted in much wider mean 90% confidence interval compared to the baseline method. We therefore choose individual scaling factors ${G}_{j}$ for each MDS coordinate.

We also use cross-validation to determine the proper smoothing for the binned kernel density estimate for the PDFs in the case of the poor-man’s approach. Specifically, the bandwidth in the kernel density method is $\mathrm{S}\times d$, where $\mathrm{S}$ is the smoothing parameter, and $d$ is the two-stage direct plug-in bandwidth estimate^57^. We select $\mathrm{S}$ for which 90% of the time the “true” changes are within the 90% confidence intervals of the kernel-smoothed PDFs obtained on the basis of the changes from the remaining models. We use 50,000 random samples during this analysis. We find that S = 2.15 is the appropriate smoothing value.

### ENSO metrics

Six metrics for monthly Niño 3 region output are chosen for projections: standard deviation, skewness, seasonality, ENSO spectrum peak, kurtosis, and lag-1 autocorrelation. Standard deviation, skewness, kurtosis, and autocorrelation are based on standard definitions. Seasonality is defined as the ratio of standard deviation in the most variable month (e.g. the month with the highest standard deviation) to the standard deviation in the least variable month. The ENSO spectrum peak is defined to be the period with the maximum spectrum density that falls within the ENSO range of 2–8 years.

## Supplementary Information

Supplementary Information.

## Data Availability

CMIP6 climate model output is publicly available from ESGF portal at https://esgf-node.llnl.gov/search/cmip6/. ERSSTv5 sea surface temperature observations can be downloaded from https://psl.noaa.gov/data/gridded/data.noaa.ersst.v5.html. SODA v2.2.4 reanalysis is publicly available online through https://iridl.ldeo.columbia.edu/SOURCES/.CARTON-GIESE/.SODA/.v2p2p4/. GODAS reanalysis output can be downloaded from https://www.cpc.ncep.noaa.gov/products/GODAS/. ORAS4 reanalysis can be obtained from https://www.cen.uni-hamburg.de/en/icdc/data/ocean/easy-init-ocean/ecmwf-ocean-reanalysis-system-4-oras4.html. HadISST observations are available from https://www.metoffice.gov.uk/hadobs/hadisst/. Multi-model projection code is publicly available from https://bitbucket.org/romanolson/probabilistic_enso_projections_public/src/master/.
