# Supplementary material for: Probabilistic projections of El Niño Southern Oscillation properties accounting for model dependence and skill
Source: Sci Rep. 2022 Dec 22;12:22128. doi: 10.1038/s41598-022-26513-3 (PMC9780329; doi:10.1038/s41598-022-26513-3)
Supplement: Supplementary file 1 — Supplementary Information. [file 41598_2022_26513_MOESM1_ESM.docx]

Supplementary Information for “Probabilistic Projections of El Niño - Southern Oscillation Properties Accounting for Model Dependence and Skill”

Roman Olson^1,2^, Soong-Ki Kim^1,3^, Yanan Fan^4^ and Soon-Il An^1,3,5,%^

^1^Irreversible Climate Change Research Center, Yonsei University, Seoul, South Korea

^2^Institude of Industrial Science, University of Tokyo, Kashiwa, Japan

^3^Department of Atmospheric Sciences, Yonsei University, Seoul, South Korea

^4^Data61, CSIRO, Australia

^5^Division of Environmental Science and Engineering, Pohang University of Science and Technology (POSTECH), Pohang, South Korea

% Corresponding author email: [sian@yonsei.ac.kr](mailto:sian@yonsei.ac.kr)

# Supplementary Note

## Validation of ENSO PDF estimation

Since ENSO PDF is highly multi-dimensional, it is difficult to gauge uncertainties inherent in its estimation in the original space directly. However, we can take advantage of a method known as multidimensional scaling (MDS) to represent the PDFs in a low-dimensional space.

The MDS takes distances between PDFs and converts them to positions in a low-dimensional space (see Methods)^1^. To test the robustness of the PDF reconstruction we input the distances between the reconstructed 50-year ENSO PDFs from 24 CMIP6 models, observations, pseudo-observations (see Methods), as well as additional second runs r2i1p1f1 for three GCMs (BCC-CSM2-MR, CAMS-CSM1-0 and INM-CM5-0) into the MDS. The 24 GCMs are chosen from a larger sample of 32 CMIP6 models as the stochastic model could not be appropriately fitted to the other models. The 102-year long pseudo-observations are simulated using the stochastic model corresponding to the observations; we then re-estimate the stochastic model from the pseudo-observations using smoothing *s*=1.5.

Here we focus on first three principal MDS dimensions. The MDS places two runs of the same GCMs close together in the configuration space (Supplementary Figure S10). In addition, the observations are placed near the pseudo-observations. This suggests that within the three principal MDS dimensions the performance of the PDF estimation method is reasonable for the datasets and the periods used here.

# References

1. Mardia, K. V. Some properties of clasical multi-dimesional scaling. *Commun. Stat. - Theory Methods* **7**, 1233–1241 (1978).

# Supplementary Figures

Figure S1: ENSO simulation in INM-CM4.8. Comparison of Niño 3 SST anomaly properties from INM-CM4.8 over years 1871-2014 (red) and from a 100,000 year long run of the fitted stochastic model (blue): (A) PDF of t [K], (B) standard deviation for different calendar months [K] and (C) spectral density [K^2^/cy mon^-1^].

Figure S2: Thermocline depth simulation in INM-CM4.8. Comparison of equatorial thermocline depth anomaly properties from INM-CM4.8 over years 1871-2014 (blue) and observations over years 1915-2016 (red): (A) PDF of h [m], (B) standard deviation for different calendar months [m] and (C) spectral density [m^2^/cy mon^-1^].

Figure S3: ENSO dynamics in INM-CM4.8 GCM. Month-on-month tendencies in Niño 3 SST anomalies [K month^-1^] as a function of equatorial thermocline depth anomalies [m] and Niño 3 SST anomalies [K] in the stochastic model constructed from INM-CM4.8 GCM for (A) March, (B) June, (C) September and (D) December. Joint thermocline depth [m month^-1^] and SST tendencies [K month^-1^] are denoted by arrows.

Figure S4: Thermocline depth simulation in INM-CM5.0. Comparison of thermocline depth anomaly properties from INM-CM5.0 over years 1871-2014 (red) and from a 100,000 year long run of the fitted stochastic model (blue): (A) PDF of h [m], (B) standard deviation for different calendar months [m] and (C) spectral density [m^2^/cy mon^-1^].

**Figure S5:** Same as figure S3 but for INM-CM5.0

**Figure S6: ENSO thermocline noise underestimation in FGOALS-g3 stochastic model.** Standard deviation of equatorial thermocline anomaly tendencies [m month^-1^] in stochastic model fitted to 1871-2014 FGOALS-g3 output (left) compared to the one fitted to observations for years 1915-2016 (right) as a function of thermocline depth anomalies [m] and Niño 3 SST anomalies [K]. Months (top to bottom): March, June, September, December.

**Figure S7:** As Figure S1, but for CanESM5.

**Figure S8: ENSO thermocline noise in CanESM5 stochastic model.** Standard deviation of equatorial thermocline anomaly tendencies [m month^-1^] in stochastic model fitted to 1871-2014 CanESM5 output as a function of thermocline depth anomalies [m] and Niño 3 SST anomalies [K] for (A) March, (B) June, (C) September, and (D) December. Compare with the right panel of Supplementary Figure S6.

**Figure S9: ENSO SST noise underestimation in FIO-ESM-2-0 stochastic model.** As Figure S6, but for Niño 3 SST anomalies from FIO-ESM-2-0 and observations.

Figure S10: Validation of the PDF estimation and MDS. Positions of PDFs derived from ENSO observations (red), alternative set of observations (pink; see Methods), pseudo-observations (orange) and CMIP6 climate models in first three principal MDS coordinates. Two runs of BCC-CSM2-MR model are shown in yellow, two runs of CAMS-CSM1-0 are shown in green, two runs of INM-CM5-0 model are shown in blue, while the rest of climate models are represented by black rectangles. R1, R2 and R3 are three first principal coordinates of the observed or modelled ENSO PDFs.
